# Decreased Pulse Wave Velocity in a Systemic Sclerosis Population: Preliminary Results from a Cross-Sectional Study

**DOI:** 10.3390/jpm12121952

**Published:** 2022-11-24

**Authors:** Francesco Salvatore Iaquinta, Roberta Grosso, Stefania Di Napoli, Velia Cassano, Saverio Naty, Giuseppe Armentaro, Mattia Massimino, Valentino Condoleo, Keti Barbara, Daniele Crescibene, Benedetto Caroleo, Sofia Miceli, Angela Sciacqua, Rosa Daniela Grembiale

**Affiliations:** 1 Department of Health Sciences, “Magna Græcia” University of Catanzaro, 88100 Catanzaro, Italy; 2 Department of Medical and Surgical Sciences, “Magna Græcia” University of Catanzaro, 88100 Catanzaro, Italy

**Keywords:** Systemic Sclerosis, arterial stiffness, pulse wave velocity, cardiovascular risk

## Abstract

Systemic Sclerosis (SSc) is an autoimmune disorder characterized by organ and tissue fibrosis in which the incidence of atherosclerosis and cardiovascular events is increased, although the exact underlying mechanism remains unclear. Arterial stiffness is a marker of vascular damage that can predict cardiovascular events; therefore, this study aimed to assess the augmentation index (AIx) and pulse wave velocity (PWV), markers of stiffness, in a Systemic Sclerosis population and to detect potentially associated variables. Fourteen female Systemic Sclerosis patients and 14 age- and sex-matched controls were enrolled. Demographic, anthropometric, sero-hematological parameters and disease characteristics were collected for each participant. Arterial stiffness was evaluated using an applanation tonometry system. No differences were found between groups, except for BMI, fasting blood glucose, red blood cells count, hemoglobin, and treatment. Patients had increased augmentation index than the controls (*p* = 0.008). PWV was significantly decreased in SSc patients compared with the controls (*p* = 0.007). PWV was correlated with age (*r* = 0.462; *p* = 0.048) and BMI (*r* = 0.458; *p* = 0.050). Finally, patients with no specific auto-antibody pattern had greater AIx than those expressing anticentromere antibodies. Our study demonstrated that SSc patients had greater AIx, but lower PWV than the controls. In addition, few variables were correlated to arterial stiffness. Further studies are necessary to validate these findings and to establish medication’s role in modifying cardiovascular risk.

## 1. Introduction

Systemic Sclerosis (SSc) is a connective autoimmune disease characterized by progressive fibrosis of several organs and tissues, more commonly affecting women [1]. Genetic and environmental factors are considered to underlie the pathophysiology of the disease by initiating a self-amplifying process that eventually leads to fibrosis of the skin and internal organs [2]. A main feature of SSc is the dysfunction of the endothelium and the consequent overproduction of the vasoconstrictor and underproduction of vasodilator factors (ET-1 and NO/prostacyclin, respectively) [3]. A common clinical manifestation is secondary Raynaud’s phenomenon (SRP), a vascular event that could result from microvasculopathy, partially due to endothelial dysfunction [4]. Furthermore, immune system activation leads to the increased production and release of pro-inflammatory cytokines, auto-antibody production, and macrophage polarization in response to endothelial cell damage and apoptosis [2]. The distinctive fibrotic process derives from multiple interactions between cells, especially fibroblasts and macrophages, and growth factors (TGF-β, PDGF), which determine the accumulation of collagen, elastin, and other extracellular matrix (ECM) proteins [5]. Thus, microvascular changes, immune system activation and fibrosis define the skin and systemic clinical manifestations of SSc [6].

In the early stage of SSc, cardiac dysfunction is frequently occult. However, structural and functional abnormalities may occur as the disease progresses; indeed, late fibrosis determines diastolic and systolic dysfunction, conduction defects, and pericardial involvement [7]. Moreover, microvascular alterations have been associated with ischemic myocardial events [8]. Although the incidence of cardiovascular (CV) events is increased in SSc [9], the exact underlying mechanisms remain unclear. A systematic review and meta-analysis of the literature [10] concluded that the risk of atherosclerosis is increased in SSc compared with a healthy population and atherosclerosis is strongly associated with inflammation, which plays a pivotal role in its initiation and progression [11]. It should be observed that inflammation in SSc is not as remarkable as in other rheumatic diseases. Therefore, unlike systemic lupus erythematosus [12] and rheumatoid arthritis [13], accelerated atherosclerosis has not yet been proven in SSc.

Arterial stiffness (AS) is known to be a marker of vascular damage and is associated with an increased risk for CV diseases (CVD), stroke, and renal disease [14]. Increased stiffness can increase the incidence of arterial obstructions as a consequence of hemodynamic changes [15]. Specifically, it increases systolic afterload, end-systolic wall stress, and finally left ventricle remodeling, while coronary perfusion pressure is reduced [16]. Carotid-femoral pulse wave velocity (cf-PWV) is the gold standard for the evaluation of arterial stiffness [17]. PWV is considered as a strong predictor of CV events or mortality, with a greater predictive value in patients with a high-risk disease state [18]. The second main indicator of arterial stiffness is the augmentation index (AIx), a composite parameter depending on PWV, which is also associated with increased risk of CV events [19,20].

Few studies have assessed arterial stiffness in SSc patients and the reported findings are contradictory. Therefore, this study aimed to define the presence of macrovascular damage in a cohort of SSc patients compared with a control group by the non-invasive measurement of arterial stiffness. Further investigations were carried out to identify worthwhile correlated variables with arterial stiffness.

## 2. Materials and Methods

### 2.1. Study Cohort

We conducted a single-center cross-sectional study analyzing the clinical, laboratory, and hemodynamic parameters of 14 consecutive Caucasian outpatients with SSc, matched for age and sex with 14 controls. Patients attending the Day Hospital of Rheumatology at the University “Magna Graecia” of Catanzaro who met the ACR/EULAR 2013 criteria for the diagnosis of SSc [21] were enrolled. All subjects aged ≥ 18 years were considered eligible for the study, while individuals with arrhythmias were excluded. At the time of enrollment, patients underwent a clinical interview, physical examination, sero-hematological features, and disease assessment including disease duration, auto-antibody pattern and current treatment. Anthropometric evaluation including height, weight, and body mass index (BMI) was performed. Traditional CV risk factors (hypertension, diabetes, dyslipidemia, smoking habit) were collected for all subjects. None had a history of angina, myocardial infarction, or valvular heart disease.

### 2.2. Laboratory Measurements

All laboratory measurements were performed after a minimum fasting period of 12 h on peripheral blood samples. Serum levels of high-sensitivity C-reactive protein (hs-CRP) were measured by the immunoturbidimetric method with an automated system (CardioPhase hsCRP, Milan, Italy). Fasting blood glucose was measured using the glucose oxidation method (Beckman Glucose Analyzer II; Beckman Instruments, Milan, Italy). Triglyceride and total, low- (LDL) and high-density lipoprotein (HDL) cholesterol concentrations were measured by enzymatic methods (Roche Diagnostics, Mannheim, Germany). Erythrocyte sedimentation rate (ESR) was determined using microphotometrical capillary stopped-flow kinetic analysis (Roller 20 LC, Alifax, Padova, Italy). Serum creatinine was evaluated using the Roche Creatinine Plus assay (Hoffman-La Roche, Basel, Switzerland). Renal function, expressed by e-GFR, was obtained according to the Chronic Kidney Disease Epidemiology (CKD-EPI) Collaboration group equation [22].

Antinuclear auto-antibody (ANA) levels were determined by indirect immunofluorescence (IIF) (Helios^®^, AESKU Diagnostics, Wendelsheim, Germany); anti-Scl70 by chemiluminescent immunoassay (CLIA) (Zenit RA Analyzer, Menarini Diagnostics, Florence, Italy); anticentromere antibodies (ACA) were detected by IIF (Helios^®^, AESKU Diagnostics, Wendelsheim, Germany), finally, anti-RNA polymerase III antibodies were determined by the ELISA kit (INOVA Diagnostics, Inc., San Diego, CA, USA). 

### 2.3. Arterial Stiffness Assessment

All subjects underwent measurements performed by the same trained physician who was blinded to the protocol. Hemodynamic data were obtained using a validated system (Sphygmocor™; AtCor Medical, Sydney, Australia) that implies a high-fidelity applanation tonometry (Millar). An integrated software directly analyzed the recorded waves. First, after 30 min of rest, blood pressure was obtained through a non-invasive, automatic recording of the brachial artery at the dominant arm (Dinamap Compact T; Johnson & Johnson Medical Ltd., Newport, UK). Measurements were obtained with the patient in the supine position, in a temperature-controlled room. The central pressure wave and pressures were directly derived from the measurement of twenty sequential radial artery waveforms using a validated transfer function [23]. Pulse wave determinations were considered reliable only if the peak and bottom variation values of the waves were <5%.

The derived systolic central wave was used to define P1, the first systolic peak due to left ventricular ejection and P2, the second systolic peak caused by pulse wave reflection. AIx was derived using the formula: (AP/pulse pressure (PP)) × 100, where the augmentation pressure (AP) is the difference between P2 and P1 and PP is the difference between the systolic and diastolic pressure. As AIx depends on the pulse wave velocity, heart rate and the amplitude of the reflected wave were recorded. The aortic cf-PWV was calculated using common carotid and femoral artery wave measurements. The following formula: PWV (m/s) = distance/time was used. Distance is the length between the carotid and femoral pulse site, whereas time refers to the wave transit. In detail, the pulse transit time from the carotid to the femoral artery was determined by measuring the R-R’ interval of an ECG performed during the exam. The distance between the sternal notch and the femoral artery was considered to define the path length between the carotid and femoral arteries. 

### 2.4. Ethical Approval

The study was approved by the local ethics committee before collecting data from the patients. Written informed consent was obtained from each participant. The Declaration of Helsinki principles were used as a guide to conduct the study. 

### 2.5. Data Analysis

Continuous variables were summarized as the mean and standard deviation (SD) (normally distributed data) or as the median and interquartile range (IQR) (non-normally distributed data), as per the Kolmogorov–Smirnov test. Categorical data were expressed as frequencies and percentages. The Mann–Whitney U, Kruskal–Wallis non-parametric test, and Chi-square test were performed to compare the variables. Among the SSc patients, linear regression analysis was used to assess the correlations between PWV and AIx with the following variables: age, body mass index (BMI), disease duration, arterial hypertension, type 2 diabetes mellitus (T2DM), dyslipidemia, total cholesterol, low-density lipoprotein, high-density lipoprotein, triglycerides, ESR, hs-CRP, endothelin receptor antagonists, calcium channel blockers, antiplatelet drug, HMG-CoA reductase inhibitors, angiotensin-converting enzyme inhibitors, angiotensin-II-receptor antagonists, steroids, β-blockers. A *p*-value ≤ 0.05 was considered to be statistically significant. IBM SPSS^®^ (IBM, Armonk, NY, USA) version 26 was used for statistical analysis.

## 3. Results

### 3.1. Study Population 

At the end of the enrollment period, 14 SSc patients and 14 sex- and age-matched controls, all females, were included in the study (Table 1). There were no significant differences in the traditional CV risk factors between the two groups. Patients with SSc had a significantly minor BMI compared to the controls (24 ± 6 vs. 26 ± 2; *p* = 0.019). The patients’ median disease duration was 11 [5.0–16] years. Concerning the sero-hematological features, the control group had a higher fasting blood glucose (FBG) than the SSc patients (107 [92–130] vs. 86 [83–96], *p* = 0.014). Moreover, the controls had a higher red blood cell (RBC) count and hemoglobin (HB) than the SSc patients (4.8 [4.4–5.3] vs. 4.3 [4.0–4.5]; *p* = 0.004 and 13 [12–14] vs. 11 [11–13]; *p* = 0.014, respectively). All 14 SSc patients were treated with iloprost and, among them, nine subjects received endothelin receptor antagonists.

Regarding the clinical and autoimmunity features of SSc patients, according to LeRoy classification [24], three patients (21%) had limited and 11 (79%) had diffuse Systemic clerosis. Antinuclear antibodies were detected in all patients. Seven patients (50%) did not show specific SSc auto-antibodies, three (21%) presented anticentromere antibodies, three (21%) showed anti Scl-70 antibodies, and only one patient (7%) expressed anti-RNA polymerase III antibodies.

### 3.2. Arterial Stiffness Assessment 

Peripheral and central hemodynamic determinations of the study population are presented in Table 2. Briefly, the measured parameters did not differ between the two groups, except for the augmentation pressure which was significantly higher in SSc patients compared with control subjects (15 [10–24] vs. 7.8 [3.7–15]; *p* = 0.035). 

**Figure 1:**
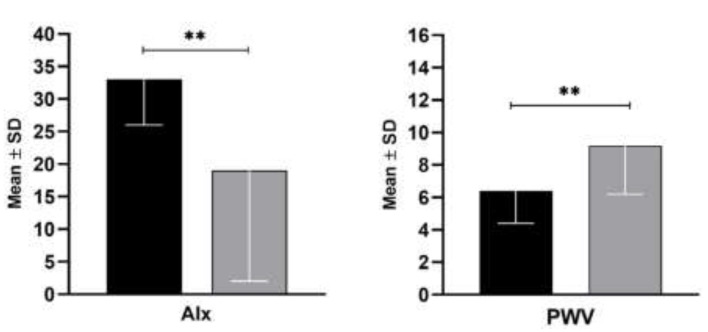
Augmentation Index (Alx) and Carotid-femoral Pulse Wave velocity (cf-PWV) in SSc patients (in black) and controls (in grey). Data are expressed as mean and standard deviation (SD). The *p*-values were calculated by the Mann-Whitney U test non-parametric test (** *p* < 0.01).

The augmentation index and cf-PWV were evaluated for each participant as previously described, and the results are presented graphically in Figure 1. As expected, the AIx was increased in the SSc patients more than in the controls (33 ± 7 vs. 19 ± 17, *p* = 0.008). Interestingly, cf-PWV was significantly reduced in the SSc patients compared with the control group (6.4 ± 2 vs. 9.2 ± 3, *p* = 0.007).

### 3.3. Arterial Stiffness-Correlated Factors

The correlation of cf-PWV and AIx with several covariates was assessed by linear regression analysis among the SSc patients (Table 3). Specifically, cf-PWV was correlated with age (*r* = 0.462; *p* = 0.048) and BMI (*r* = 0.458; *p* = 0.050).

AIx differed among the disease auto-antibodies (Table 4). Namely, patients negative for specific antibodies had increased AIx compared with patients expressing anticentromere antibodies. Moreover, concerning SSc cutaneous subsets, AIx and PWV were slightly increased in the lcSSc patients (*p* = 0.885 for each).

## 4. Discussion

Arterial stiffness has been widely correlated with aging, but also with arterial hypertension and diabetes. The underlying process includes a complex interplay between factors, among which are collagen-elastin and metalloprotease (MMP) balance as well as endothelial dysfunction [25].

In this cross-sectional study, we evaluated arterial stiffness in a cohort of 14 female Caucasian SSc patients and 14 female control subjects. The groups did not differ for traditional CV risk factors, and only BMI was slightly lower in SSc patients than in the controls (24 ± 6 vs. 26 ± 2; *p* = 0.019). Our study showed that patients with SSc had increased AIx compared with the controls (33 ± 7 vs. 19 ± 17, *p* = 0.008), but not cf-PWV, which was surprisingly lower (6.4 ± 2 vs. 9.2 ± 3, *p* = 0.007).

In the previous literature, uneven results have been reported. A study conducted by Timar et al. [26] demonstrated that both AIx and PWV were increased in SSc patients compared with a healthy control group, the authors also noted a significant correlation between AS, age, and disease duration. Similar findings have been reported in other studies. In more detail, Sunbul et al. [27] evaluated AS by the oscillometric method and showed that patients with SSc had higher arterial stiffness parameters than the controls, but consistently with our results, AIx and PWV did not differ when the SSc patients were divided into two groups according to the cutaneous subset. In the study performed by Colaci et al. [28], PWV was found to be increased in SSc patients compared with 26 controls, however, the increase in PWV was observed only in patients > 50 years old and no data were provided for the augmentation index. Conversely, in the observational study of G.-S. Ngian et al. [29], AIx was higher in the SSc patients, whereas, although PWV was increased, it did not reach statistical significance. Other studies revealed similar results [30,31]. 

PWV values play a crucial role in predicting CV events and mortality. Specifically, an increase of 1 m/s of PWV determines a risk increase of total CV events by 14%, CV mortality by 15%, and all-cause mortality by another 15% [32].

To our best knowledge, this is the first study to report a decrease in PWV in SSc patients. However, it should be considered that while PWV is a direct parameter of arterial stiffness, AIx depends on three factors: heart rate, PWV, and the amplitude of the reflected wave. Thus, an increased AIx might be the expression of peripheral microvascular disease, a main hallmark of SSc, suggesting that the small arteries are involved earlier than the large ones. Moreover, we found few correlations between PWV and the variables studied. Specifically, PWV was associated with age (*r* = 0.462; *p* = 0.048) and BMI (*r* = 0.458; *p* = 0.050). The reported data are in line with the current knowledge defining age and BMI as major determinants of arterial stiffness. 

No correlations were found with treatment, although calcium channel blockers and ACE inhibitors resulted in being close to statistical significance (*p* = 0.052 and *p* = 0.057; respectively) for correlation with PWV. It is worth noting that the analysis was conducted solely on the 14 patients enrolled in the study, therefore, a larger sample size may detect the correlations more effectively. In a previous study [29], CCB treatment was reported to be directly associated with AIx, while we found a non-significative negative correlation with PWV. Long-term treatment with CCB can decrease the arterial stiffness and prevent its progression by reducing aortic wall remodeling. Moreover, nifedipine has been reported to increase endothelial nitric oxide production [33], which is impaired in SSc. Recently updated guidelines on SSc treatment recommend CCBs as first-line therapy for Raynaud’s phenomenon [34]. It must be observed that in our study, all 14 patients were treated with iloprost, which is a potent vasodilator and may impact, to some extent, the arterial stiffness. However, so far, its role has not been investigated. In a trial [35], the prostacyclin analogue beraprost has been shown to prevent the development of arterial stiffness in patients with cerebral infarction. The role of prostacyclin analogues cannot be excluded; thus, it needs to be investigated more carefully.

The current study has some limitations. The AS parameters were statistically different between the two groups, however, only a few correlations with the demographic and clinical variables were found. This could be explained by the limited number of enrolled subjects; therefore, a larger sample size will be necessary to detect further associations. Moreover, as the cross-sectional design of the study prevented the definition of causality, longitudinal protocols should be carried out to better define and investigate the predictive value of arterial stiffness for cardiovascular events in SSc patients as well as the role of treatment in modifying AS measurements. Finally, this was a single-center study including all-female Caucasian patients, therefore, generalization of the findings remains to be determined.

## 5. Conclusions

In conclusion, our results support the evidence of an increased AIx, but not PWV, in SSc patients. In addition to the correlations found for PWV with both age and BMI, treatment might have some impact on the AS of these patients, therefore, an in-depth analysis is required. Arterial stiffness measurement may be a reliable and reproducible method for identifying macrovascular involvement in patients with SSc, being relevant in the clinical setting due to its predictive value of CV events.

## Figures and Tables

**Table 1:** Characteristics of the study population.

	Controls (n = 14)	SSc Patients (n = 14)	*p*
**Demographic and Anthropometric**			
Age (years)	60 ± 4	61 ± 13	0.836
Gender, female	14 (100)	14 (100)	-
BMI (kg/m^2^)	26 ± 2	24 ± 6	**0.019**
Disease Duration (years)	-	11 [5.0–16]	-
**Risk Factors**			
Smoking habit, yes (%)	5 (36)	5 (36)	0.999
AH, yes (%)	7 (50)	8 (57)	0.705
T2DM, yes (%)	5 (36)	4 (29)	0.686
Dyslipidemia, yes (%)	6 (43)	4 (29)	0.430
CVD familiarity, yes (%)	6 (43)	4 (29)	0.430
**Sero-hematological** **characteristics**			
Total Cholesterol (mg/dL)	189 [167–218]	170 [128–197]	0.094
LDL (mg/dL)	117 [96–139]	100 [80–115]	0.125
HDL (mg/dL)	61 [50–77]	56 [45–63]	0.329
Triglycerides (mg/dL)	107 [62–133]	107 [69–151]	0.667
FBG (mg/dL)	107 [92–130]	86 [83–96]	**0.014**
ESR (mm/h)	22 [18–22]	18 [7.5–27]	0.541
CRP (mg/L)	3.2 [2.0–5.3]	3.2 [3.2–10]	0.227
RBC (×10^3^/μL)	4.8 [4.4–5.3]	4.3 [4.0–4.5]	**0.004**
WBC (×10^6^/μL)	6.2 [5.9–8.5]	6.4 [5.1–7.6]	0.454
HB (g/dL)	13 [12–14]	11 [11–13]	**0.014**
PLT (×10^3^/μL)	245 [204–293]	223 [187–242]	0.164
Creatinine (mg/dL)	0.71 [0.59–0.88]	0.72 [0.64–0.99]	0.511
e-GFR	93 [69–103]	94 [56–100]	0.701
**Treatment**			
Iloprost, yes (%)	0 (0)	14 (100)	**1.21 × 10^−7^**
ERA, yes (%)	0 (0)	9 (64)	**2.71 × 10^−4^**
CCB, yes (%)	8 (57)	4 (29)	0.127
Β-blockers, yes (%)	0 (0)	5 (36)	**0.014**
Antiplatelet drug, yes (%)	8 (57)	10 (71)	0.430
HMG-CoA reductase inhibitors, yes (%)	6 (43)	4 (29)	0.430
ACE inhibitors, yes (%)	2 (14)	6 (43)	0.094
Angiotensin-II-receptor antagonists, yes (%)	0 (0)	2 (14)	0.142
Steroids, yes (%)	0 (0)	7 (50)	**0.002**

Abbreviations: SSc: Systemic Sclerosis; BMI: body mass index; AH: arterial hypertension; T2DM: Type 2 diabetes mellitus; CVD: cardiovascular disease; LDL: low-density lipoprotein; HDL: high-density lipoprotein; FBG: fasting blood glucose; ESR: erythrocyte sedimentation rate; CRP: C-reactive protein; RBC: red blood cell; WBC: white blood cell; HB: hemoglobin; PLT: platelet; e-GFR: estimated glomerular filtration rate; ERA: endothelin receptor antagonists; CCB: calcium channel blockers; ACE inhibitors: angiotensin-converting enzyme inhibitors.

**Table 2:** Peripheral and central hemodynamic determinations of the study group.

Variables	Controls (n = 14)	SSc Patients (n = 14)	*p*
HR, bpm	68 [63–73]	70 [67–77]	0.541
sBP, mmHg	128 [112–143]	123 [119–147]	0.603
dBP, mmHg	74 [60–87]	77 [73–80]	0.482
PP, mmHg	51 [45–62]	47 [44–67]	0.946
Central-sBP, mmHg	122 [111–142]	117 [113–134]	1
Central-dBP, mmHg	78 [72–84]	79 [75–82]	0.635
Central-PP, mmHg	44 [38–61]	41 [35–57]	0.635
AP	7.8 [3.7–15]	15 [10–24]	0.035

Abbreviations: SSc: Systemic Sclerosis; HR: heart rate; sBP: systolic blood pressure; dBP: diastolic blood pressure; PP: pulse pressure; AP: augmentation pressure.

**Table 3:** Correlations between the variables and arterial stiffness parameters in the SSc patients.

	Augmentation Index		cf-Pulse Wave Velocity	
	*r*	*p*	*r*	*p*
Age	0.349	0.111	**0.462**	**0.048**
BMI	−0.375	0.093	**0.458**	**0.050**
Disease duration	0.061	0.418	−0.124	0.336
AH	0.270	0.176	0.318	0.134
T2DM	0.125	0.336	−0.006	0.492
Dyslipidemia	0.010	0.487	−0.078	0.396
Total Cholesterol	−0.414	0.070	0.151	0.304
LDL	−0.225	0.220	0.195	0.252
HDL	−0.315	0.136	0.205	0.241
Triglycerides	−0.370	0.096	−0.139	0.318
ESR	0.064	0.414	−0.131	0.328
CRP	0.123	0.338	−0.091	0.378
ERA	0.037	0.450	−0.376	0.093
CCB	0.262	0.182	−0.453	0.052
Antiplatelet drug	0.082	0.390	0.166	0.286
HMG-CoA reductase inhibitors	0.010	0.487	−0.078	0.396
ACE inhibitors	0.171	0.280	0.441	0.057
Angiotensin-II-receptor antagonists	0.140	0.317	−0.370	0.096
Steroids	−0.104	0.362	−0.426	0.064
β-blockers	0.244	0.200	0.150	0.305

Abbreviations: SSc: Systemic Sclerosis; BMI: body mass index; AH: arterial hypertension; T2DM: Type 2 diabetes mellitus; LDL: low-density lipoprotein; HDL: high-density lipoprotein; ESR: erythrocyte sedimentation rate; CRP: C-reactive protein; ERA: endothelin receptor antagonists; CCB: Calcium channel blockers; ACE inhibitors: angiotensin-converting enzyme inhibitors.

**Table 4:** Arterial stiffness measurement in the clinical and serological subtypes of SSc patients.

	Negative (n = 7)	ACA (n = 3)		Scl70 (n = 3)	*p*
AIx	37 ± 5	26 ± 2		28 ± 4	0.040 *
cf-PWV	7 ± 2	7 ± 2		5 ± 1	0.234
	**lcSSc** **(n = 3)**		**dcSSc** **(n = 11)**		***p***
AIx	34 ± 5		33 ± 8		0.885
cf-PWV	7 ± 3		6 ± 2		0.885

Abbreviation: AIx: augmentation index; cf-PWV: carotidal-femoral pulse wave velocity; ACA: anticentromere antibodies; RNAPIII: anti-RNA polymerase III antibodies; Scl70: anti-topoisomerase I antibodies; lcSSc: limited cutaneous Systemic Sclerosis; dcSSc: diffuse cutaneous Systemic Sclerosis. * Negative vs. ACA: *p* = 0.020.

## Data Availability

The data presented in this study are available on request from the corresponding author. The data are not publicly available due to the sensitive information in the database.
